# Frozen-thawed double cleavage-stage or frozen-thawed single day 6 blastocyst stage embryo transfer: which is preferable for patients younger than 35 without day 5 blastocyst formation?

**DOI:** 10.3389/fendo.2025.1473854

**Published:** 2025-05-14

**Authors:** Yan Liu, Taojun Wang, Shanjun Dai, Hao Shi, Yingpu Sun

**Affiliations:** ^1^ Center for Reproductive Medicine, The First Affiliated Hospital of Zhengzhou University, Zhengzhou, Henan, China; ^2^ Henan Key Laboratory of Reproduction and Genetics, The First Affiliated Hospital of Zhengzhou University, Zhengzhou, Henan, China

**Keywords:** cleavage-stage embryo transfer, blastocyst-stage embryo transfer, single embryo transfer, transfer strategy, frozen-thawed transplantation

## Abstract

**Background:**

To compare the clinical outcomes between frozen-thawed cleavage embryo transfer and frozen-thawed day 6 blastocyst transfer in patients younger than 35 using the freeze-all strategy without day 5 blastocyst formation.

**Methods:**

This was a retrospective observational analysis performed between January 2018 and December 2022 at the Reproductive and Genetic Specialist Hospital of the First Affiliated Hospital of Zhengzhou University. A total of 576 patients younger than 35 who used the “freeze-all strategy” but produced no day 5 blastocysts were recruited. The patients were divided into 3 groups according to the number and stage of the transferred embryos: double cleavage-stage embryos (Group A), single good-quality day 6 blastocysts (Group B) and single inferior-quality day 6 blastocysts (Group C),and several pregnancy outcomes were measured.

**Results:**

Groups A and B exhibited significantly higher chemical (73.7%, 67.0% versus 51.9%) and clinical pregnancy rates (69.0%, 59.4% versus 44.2%) than Group C. The implantation rate was significantly higher in Group B than in Groups A and C (59.4% versus 45.7%, 43.5%). The live birth rate was significantly higher in Group A than in Group C (59.2% versus 48.1%). The multiple pregnancy rate was significantly higher in Group A than in Groups B and C (34.4% versus 1.6%, 1.5%). The early miscarriage rate was significantly higher in Group C than in Group A and Group B (23.5% versus 8.7%, 12.7%). Premature delivery rates, late miscarriage rates and ectopic pregnancy rates were comparable across groups.

**Conclusions:**

A single good quality day 6 blastocyst transfer was the preferable strategy for the freeze-all strategy patients who younger than 35 and without day 5 blastocyst formation.

## 1 Introduction

Over the past several decades, with the extension of embryo culture duration *in vitro* (1, 2) and the broad application of vitrification (3, 4), single blastocyst transfer has been increasingly applied in the clinical setting to avoid ovarian hyperstimulation syndrome (OHSS) and multiple pregnancy (5–8).

Whether to transfer cleavage-stage or blastocyst-stage embryos has become a controversial question in recent years. Some studies suggest that blastocyst-stage embryo transfer is more efficient (9, 10). First, the extended culture of the embryo *in vitro* promotes embryo self-selection; that is, only embryos with good developmental potential are capable of forming blastocysts (11). Second, in naturally occurring pregnancies, embryo implantation occurs at the hatched-blastocyst stage; therefore, blastocyst-stage embryo transfer may better mimic the physiologic timing of exposure of the embryo to the uterine environment (12). Third, some studies have suggested that blastocyst-stage embryo transfer achieves significantly higher implantation, clinical pregnancy, and live birth rates and comparable miscarriage rates compared with cleavage-stage embryo transfer in high responders (13).

Conversely, some studies indicate that blastocyst-stage embryo transfer is not superior to cleavage-stage embryo transfer (14). First, only 60~80% of cleavage-stage embryos can progress to blastocyst-stage embryos due to self-selection, which may result in a higher incidence of cycle cancellation and lower rates of embryo cryopreservation. Second, some studies have shown that there is no significant difference in live birth, ongoing pregnancy, clinical pregnancy or miscarriage rates between transfers using embryos in these two stages. Third, the extension of the culture period is more time consuming and costly, and two to four days are required for cleavage-stage embryos to develop to blastocyst-stage embryos (15, 16).

Most studies in recent years have indicated that day 5 blastocysts have a higher euploidy rate than day 6 blastocysts (17, 18). Day 5 blastocyst transfer is generally considered to have a higher implantation rate, clinical pregnancy rate and live birth rate than day 6 blastocyst transfer (19), but a limitation of previous studies is that they usually only compared the clinical outcomes within blastocyst-stage and cleavage-stage embryos and did not differentiate between blastocysts at different days of development or between transfers of different numbers of blastocysts. Most embryologists prefer single day 5 blastocyst-stage embryo transfer to achieve higher clinical pregnancy and a lower multiple pregnancy rates, but if patients have no day 5 blastocyst-stage embryos cryopreserved, whether double cleavage-stage embryo transfer or single day 6 blastocyst-stage embryo transfer is more likely to achieve a satisfactory clinical outcome is still a matter of debate.

The aim of this study is to compare the clinical outcomes between frozen-thawed cleavage embryo transfer and frozen-thawed day 6 blastocyst transfer in patients younger than 35 using the freeze-all strategy without day 5 blastocyst formation. We concluded that single good quality day 6 blastocyst transfer was the preferable strategy with a comparable clinical outcomes and significantly lower multiple pregnancies compared with the double cleavage embryo transfer group. This conclusion can give a suggestion for embryologists to provide a more effective selection for the stage of the transferred embryos for who had no day 5 blastocyst cryopreservation.

## 2 Materials and methods

### 2.1 Patients and groups

This study was approved by the Ethics Committee of the First Affiliated Hospital of Zheng Zhou University. The data of 29297 couples treated in the Reproductive Medical Center of the First Affiliated Hospital of Zhengzhou University between January 2018 and December 2022 were collected. Eventually, 576 couples were included and analyzed in accordance with the inclusion criteria and exclusion criteria. This study enrolled only patients under 35 years who were undergoing their first frozen-thawed cycle and had at least two cleavage-stage embryos and one blastocyst-stage embryo available at the same time.

**Figure 1:**
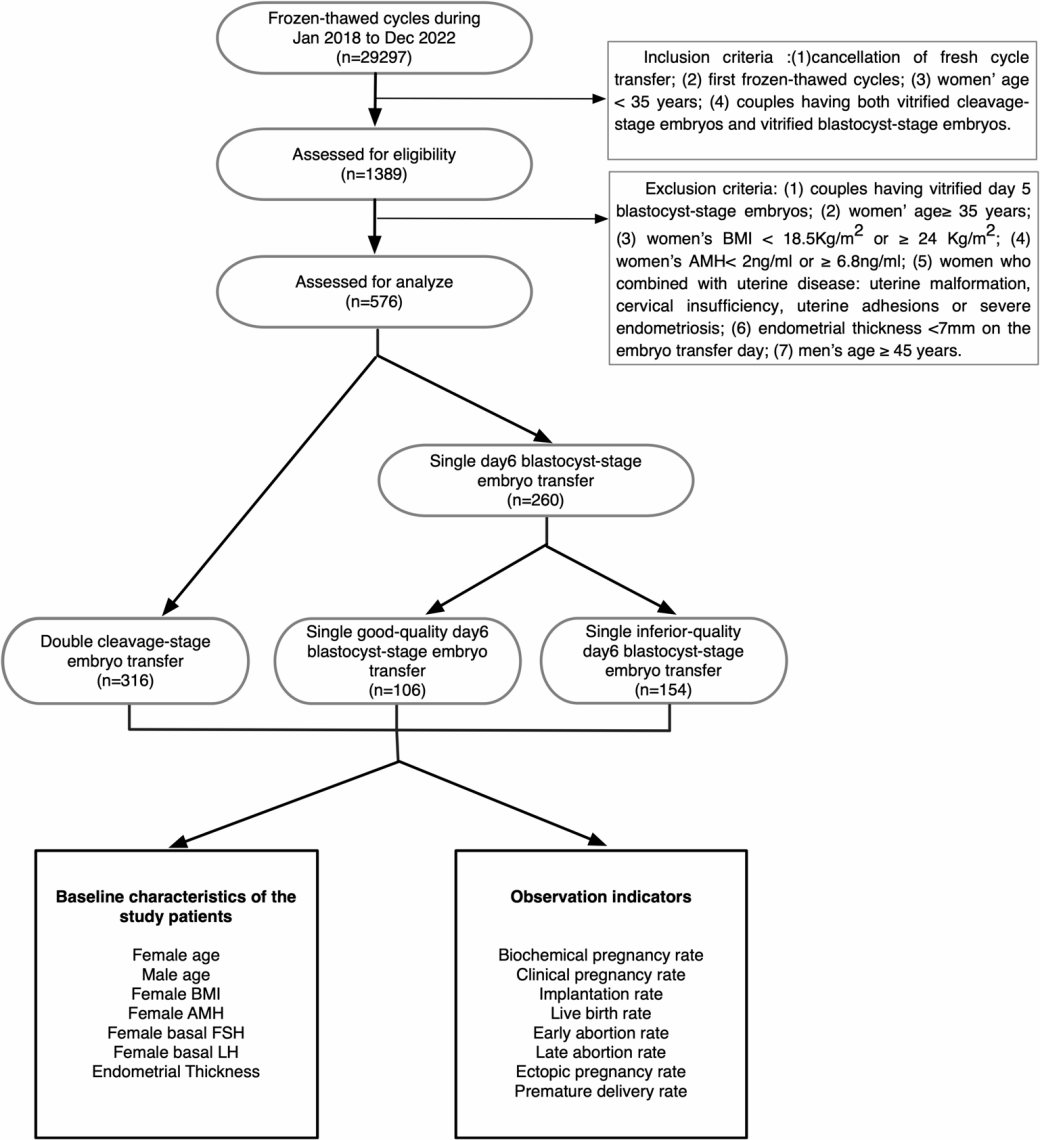
Flow diagram showing patient allocation.

The 576 couples were divided into three groups according to the stages and the numbers of embryos transferred: double cleavage-stage embryos (Group A), single good-quality day 6 blastocyst (Group B) and single inferior-quality day 6 blastocyst (Group C). The route of our study is shown in **Figure 1**.

### 2.2 Inclusion criteria and exclusion criteria

The inclusion criteria were as follows: (1) cancellation of fresh cycle transfer; (2) first frozen-thawed cycle; (3) female partner aged < 35 years on the day of embryo transfer; and (4) couples having both vitrified cleavage-stage embryos and vitrified blastocyst-stage embryos.

The exclusion criteria were as follows: (1) couples with vitrified day 5 blastocyst-stage embryos; (2) female partner aged ≥ 35 years on the day of embryo transfer; (3) female partner’s body mass index (BMI) < 18.5 kg/m^2^ or ≥ 24 kg/m^2^; (4) anti-Mullerian hormone (AMH) < 2 ng/ml or ≥ 6.8 ng/ml; (5) uterine malformation, cervical insufficiency, uterine adhesions or severe endometriosis; (6) endometrial thickness <7 mm on the day of embryo transfer; and (7) male partner aged ≥ 45 years on the day of embryo transfer.

### 2.3 Vitrification, thawing and embryo culture

Vitrification of both cleavage- and blastocyst-stage embryos was performed following the manufacturer’s instructions (Vitrification Kit, Kitazato, Japan). The thawing process also followed the manufacturer’s instructions (Thawing Kit, Kitazato, Japan). The thawed cleavage or blastocyst embryos were transferred into overnight balanced G-2 Plus (G-2 plus, Vitrolife, Sweden), and every thawed embryo was cultured in overnight balanced G-2 Plus at 37°C and 6% CO_2_ at least 2 hours before embryo transfer.

### 2.4 Morphological grading of embryos

Cleavage scoring was performed according to the Peter scoring standard (20); the number of blastomeres is more than 6, the size of blastomeres is slightly uneven, and the cell fragments are < 10% were considered high-quality cleavage-stage embryos. Only high-quality cleavage-stage embryos were cryopreserved. In this study, post-thaw survival for cleavage-stage embryos was defined as more than half of the original cells remaining intact and at least four blastomeres.

The blastocysts were evaluated using the Gardner blastocyst scoring system (12), and the post-thaw survival of cryopreserved blastocyst-stage embryos was defined as maintenance of expansion ability. A surviving blastocyst was considered worth transferring if the degree of blastocyst expansion was at least ≥3, the inner cell mass (ICM) was at least grade B and the trophectoderm (TE) was at least grade C. In this study, blastocysts of 3BB and above (grade C without ICM or TE) were defined as good-quality and those with grade C TE were defined as inferior quality.

Both the cleavage scoring and the blastocyst scoring were scored according on the state of embryos before cryopreservation.

### 2.5 Endometrial preparation

The endometrial preparation plan was selected as appropriate according to the patient’s condition: 1) For patients with a regular menstrual cycle, follicular development and endometrial condition were monitored by transvaginal ultrasound on the 10-12^th^ day of the menstrual cycle. When the diameter of the dominant follicle was between 18–20 mm and a urinary LH (Luteinizing Hormone, LH) peak appeared, or if there was no LH surge, 10000 IU of human chorionic gonadotropin (HCG, Zhuhai,Lizhu) was given to induce ovulation in the morning in order to control the ovulation time. An endometrial thickness ≥7 mm was considered appropriate for embryo transfer. 2) Artificial cycle: Estradiol valerate tablets (Estradiol Valerate, Bayer, Germany) were given 2–4 mg/d on the 2^nd^-3^rd^ day of the menstrual period or progesterone withdrawal bleeding, the endometrial thickness was monitored by ultrasound, and the dosage of Estradiol valerate tablets was adjusted according to the serum estrogen level and endometrial condition.

### 2.6 Pregnancy outcome evaluation

Fourteen days after embryo transfer, the serum β-human chorionic gonadotropin (HCG) level was detected to determine whether pregnancy had been established. Chemical pregnancy was defined as β-HCG > 5 U/mL. Clinical pregnancy was diagnosed as the presence of a gestational sac and primitive heart tube pulsation on transvaginal ultrasound examination 35 days after embryo transfer, and multiple pregnancy was judged according to the number of sacs present. If the β-HCG-positive gestational sac was not in the normal position in the uterine cavity, it was considered as ectopic pregnancy; spontaneous abortion was diagnosed if the embryo stopped developing or the fetus had no heartbeat at a later follow-up. Luteal support drugs were given when a pregnancy was confirmed and discontinued in cases of pregnancy loss.

### 2.7 Outcome indicator calculations

The calculations of the outcome indicators were as follows:

1. Biochemical pregnancy rate = number of biochemical pregnancy cycles/number of transplantation cycles × 100%;
2. Clinical pregnancy rate = number of clinical pregnancy cycles/number of transplantation cycles × 100%;
3. Implantation rate = number of gestational sacs/number of transplanted embryos × 100%;
4. Live birth rate = number of live birth cycles/number of transplantation cycles × 100%;
5. Early abortion rate = number of spontaneous abortions before 12 weeks of pregnancy/number of clinical pregnancy cycles × 100%;
6. Late abortion rate = number of spontaneous abortions from 12 to 28 weeks of pregnancy/number of clinical pregnancy cycles × 100%;
7. Ectopic pregnancy rate = number of ectopic pregnancy cycles/number of clinical pregnancy cycles × 100%;
8. Premature delivery rate = number of live births at 28–36 gestational weeks/number of live birth cycles × 100%.

### 2.8 Statistical analyses

SPSS 21.0 software (IBM, USA) was used to process the data, and the quantitative data are expressed as the mean standard deviation (X ± S). The independent samples T test was used for comparisons between two groups, and one-way ANOVA was used for comparisons between multiple groups. Univariable logistic regressions were used to analyze the correlation of the three transfer groups with the clinical outcomes. Qualitative data were expressed as percentages (%) and compared using the chi-square test or Fisher’s exact probability method; differences were considered statistically significant at p< 0.05.

## 3 Results

### 3.1 Study population and cycle characteristics

In total, 892 frozen-thawed embryos from 576 patients were retrospectively analyzed (see **Table 1** for basic data and cycle characteristics of the patients in the three groups). There were no significant differences among these three groups in terms of male age (years), female BMI (kg/m^2^), female AMH (ng/ml), female basal FSH (mIU/ml), female basal LH (mIU/ml) or endometrial thickness on transplantation day (mm). The distribution of the endometrial preparation protocols in these three groups was not significantly different, but the female age (years) in Group A was significantly younger compared with the other groups(28.61 ± 3.15 versus 29.42 ± 2.97, 29.51 ± 3.17, p<0.05).

**Table 1:** Basic data and cycle characteristics of the patients in the three groups.

		Group A	Group B	Group C	P value
No. of frozen-thawed cycles		316	106	154	
Average no. of transferred embryos		2	1	1	
Female age (years)		28.61 ± 3.15	29.42 ± 2.97	29.51 ± 3.17	*p*>0.05^c^,p<0.05^ab^
Male age (years)		29.99 ± 4.01	29.98 ± 3.23	30.37 ± 3.62	*p*>0.05^abc^
Female BMI (kg/m^2^)		21.30 ± 1.52	21.39 ± 1.52	21.42 ± 1.48	*p*>0.05^abc^
Female AMH (ng/ml)		3.98 ± 1.29	3.80 ± 1.29	3.86 ± 1.25	*p*>0.05^abc^
Basal FSH (mIU/ml)		6.21 ± 1.37	6.48 ± 1.43	6.53 ± 1.82	*p*>0.05^abc^
Basal LH (mIU/ml)		5.95 ± 5.77	5.94 ± 5.66	8.70 ± 14.38	*p*>0.05^abc^
Endometrial thickness on transplantation day (mm)		10.47 ± 1.72	10.82 ± 1.99	10.70 ± 1.75	*p*>0.05^abc^
Endometrial preparation	E-P protocol	42.41% (134/316)	46.22% (49/106)	40.91% (63/154)	*p*>0.05^abc^
	Natural protocol	49.68% (157/316)	48.11% (51/106)	52.59% (81/154)	*p*>0.05^abc^
	others	7.91% (25/316)	5.67% (6/106)	6.49% (10/154)	*p*>0.05^abc^

No, Number; BMI, Body mass index; AMH, Anti-Mullerian hormone; E-P, Estradiol-progesterone. Values are presented as the mean ± standard deviation (SD) or number (%, number).

a, Group A versus Group B; b, Group B versus Group C, Group A versus group C.

### 3.2 The comparison of the clinical outcomes between the three transfer groups

**Figure 2:**
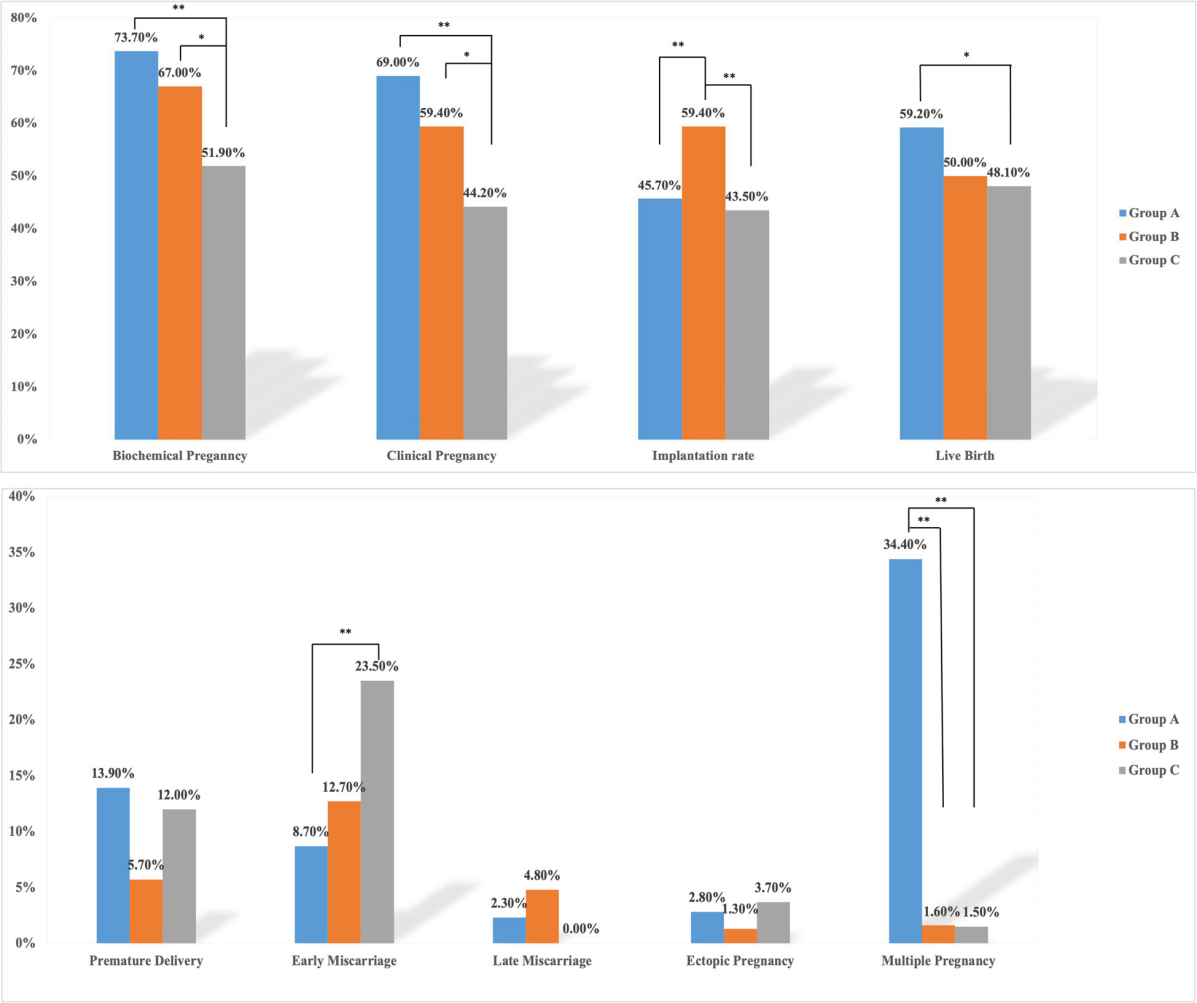
Rates of each outcome in the three groups. Group A, double cleavage-stage embryo transfer group; Group B, single day 6 good-quality blastocyst-stage embryo transfer group; Group C, single day 6 inferior-quality blastocyst-stage embryo transfer group. *denotes *p*<0.05, **denotes *p*<0.01. The blue bar chart represents Group A; the orange bar chart represents Group B; the grey bar chart represents Group C.

Group A and Group B had significantly higher chemical pregnancy and clinical pregnancy rates compared with Group C (73.7%, 67.0% versus 51.9%, p<0.05; 69.0%, 59.4% versus 44.2%, p<0.05). Group B had a significantly higher implantation rate than Group A and Group C (59.4% versus 45.7%, 43.5%, p<0.05). Group A had a significantly higher live birth rate than Group C (59.2% versus 48.1%, p<0.05), but the differences between Group A and Group B and between Group B and Group C were not significant. Group C had a significantly higher early miscarriage rate than Group A and Group B (23.5% versus 8.7%, 12.7%, p<0.05); no significant difference were found in late miscarriage rate and ectopic rate between these three groups. Group A had a significantly higher multiple pregnancy rate than Group B and Group C (34.4% versus 1.6%, 1.5%, p<0.05). Interestingly, the premature delivery rate was apparently higher in Group A than in Group B or C (13.9% versus 5.67%, 12.0%), but the differences were not statistically significant (**Figure 2**).

### 3.3 The correlation of the three transfer groups with the clinical outcomes

In order to compare the efficacy of the different transfer strategies, logistic regression analysis was conducted to detect the association of the clinical outcomes within the three transfer groups (**Table 2**), adjusted OR were analyzed due to the significantly differences in female ages. From the **Table 2**, no statistical significant difference was detected with the clinical pregnancy rate, live birth rate, abortion rate between the group A and group B. There were a lower probability of clinical pregnancy (aOR 0.501, 95%CI 0.335-0.749) and live birth (aOR 0.337, 95%CI 0.223-0.509) in the transfer of the Group C compared with the transfer of the Group A, but there was a higher risk of abortion (aOR 2.477, 95%CI 1.198-5.122) in the transfer of the Group C.

**Table 2:** Logistic regression analysis of pregnancy, live birth and abortion outcomes between the three groups.

Groups	Clinical pregnancy			Live birth			Abortion			
	P value	OR	95% CI	P value	OR	95% CI	P value	OR	95% CI
Group A	0.001			0.000			0.034		
Group B	0.072	0.659	0.418-1.038	0.042	0.631	0.406-0.983	0.176	1.710	0.787-3.717
Group C	0.000	0.486	0.327-0.722	0.000	0.327	0.218-0.491	0.011	2.487	1.232-5.022
Intercept	0.000	2.224		0.001	1.469		0.000	0.124	
	Pvalue	aOR	95% CI	Pvalue	aOR	95% CI	Pvalue	aOR	95% CI
Group A	0.003			0.000			0.047		
Group B	0.088	0.665	0.417-1.062	0.067	0.653	0.413-1.030	0.243	1.630	0.718-3.703
Group C	0.001	0.501	0.335-0.749	0.000	0.337	0.223-0.509	0.014	2.477	1.198-5.122
Intercept	0.061	18.950		0.007	63.926		0.066	0.006	

CI, confidence interval; OR, odds ratio^a^ adjusted ORs were obtained using univariate regression after controlling for the following confounding factors: the female age on the day of the transfer, the male age on the day of the transfer, the female BMI, the female AMH, the female bFSH, the female bLH, Endometrial thickness on transplantation day.

## 4 Discussion

The cleavage-stage embryo transfer or the day 6 blastocyst-stage embryo transfer is seldom studied in the couples who had no day 5 blastocyst cryopreservation over the past two decades. In this retrospective study, we confirmed that good-quality blastocysts, even day 6 blastocysts, had a significantly higher implantation rate than cleavage-stage embryos and poor-quality day 6 blastocysts, but the implantation rate of poor-quality day 6 blastocysts was comparable to that of cleavage-stage embryos. This conclusion is consistent with those of most studies published in recent decades (21). The main reason for this may be that there is a positive relationship between blastocyst formation and embryo euploidy (20–22), such that cleavage-stage embryos with poor developmental potential often fail to develop into blastocysts (23, 24). In addition, the timing of blastocyst transfer is identical to that in natural pregnancy and synchronized with the endometrial environment (25, 26). Some papers proved that the implantation rate of blastocysts is related not to the developmental stage of the blastocysts but rather to TE quality, which may be because the euploidy rate of blastocysts with a poor TE grade was significantly lower than that of those with a high-quality TE (27–29).

We found that although good-quality day6 blastocyst had a significantly higher implantation rate, the double cleavage-stage embryo transfer can achieve a comparable chemical pregnancy and clinical pregnancy rates compared with to those of a single good-quality day 6 blatstocyst transfer. the main reason for this maybe the increased number of transferred embryos can correspondingly increase the probability of a positive clinical outcome of the embryo transfer, A meta-analysis showed that double cleavage transfer had a apparently higher clinical pregnancy rate and ongoing pregnancy rate compared with the single cleavage transfer (30). In a randomized controlled trial from Aafke also showed in unselected patient, double embryo transfer can resulted in significantly higher pregnancy rates compared with the elective single embryo transfer (31).

Although double cleavage-stage embryo transfer achieved a comparable clinical pregnancy rates and live birth rates compared with single good-quality blastocyst-stage embryo transfer in our study, it was also associated with significantly higher multiple pregnancy rates compared with the other groups. Multiple pregnancy is also associated with a significantly higher risk of perinatal mortality and morbidity, preterm birth, neonatal death and maternal complications such as hypertensive disorders, gestational diabetes and postpartum hemorrhage (32–37). In addition, multiple pregnancy is also associated with social, financial, and psychological implications for new parents, with higher levels of stress and lower quality of life (38). So in recent years, embryologists have focused on selective single embryo transfer (sSET) to reduce multiple pregnancy rates while ensuing stable pregnancy and live birth rates. Some studies showed that in good-prognosis patients or the females under 35 years old who had normal menstrual cycles, single day 5 blastocyst transfer or even a selected single cleavage-stage embryo transfer can achieve a comparable clinical pregnancy rates, live birth rates and cumulative pregnancy rates compared with double embryo transfer (39, 40). Moreover, although the premature delivery rates were not significantly different among these three groups in our data, the highest premature delivery rate was observed for the double cleavage-stage embryo transfer group. Many previous studies have shown that an increased number of transferred embryos is a relevant risk factor for premature delivery (41, 42). The lack of statistical difference in our study may be due to the limited sample size. The multiple pregnancy rates in the two blastocyst-stage transfer groups were not significantly different, besides both of them were significantly lower than the double cleavage-stage transfer group, so it is suggesting that it is the increased number of transferred embryos, not the stage of the transferred embryos is the relevant risk factor for multiple pregnancy.

From our data, it was showed that patients receiving poor-quality day 6 blastocysts had a significantly higher early miscarriage rate than those in the other groups, but the late miscarriage rates and ectopic pregnancy rates were comparable among the three groups. It is believed that higher trophoblast (TE) morphology scores correlate with higher euploidy rates and higher pregnancy rates (43–45), and at present, the evaluation of embryos mainly relies on morphological scores, but some studies demonstrated that even some aneuploidy embryos can also eventually develop into high-quality blastocysts (46). but some papers have showed that in the case of blastocysts transferred after confirmation of euploidy by preimplantation genetic testing (PGT), there is no difference in implantation rate among blastocysts of different development days and different grades; the implantation rate of even poor-quality biopsied blastocysts did not differ significantly from that of high-quality blastocysts (17–27). In the past, it was showed that the prevalence of chromosome abnormalities in women experiencing early miscarriage was as high as 45% (46–48), and the detection of submicroscopic chromosome anomalies in miscarriage samples using molecular techniques has suggested that more than 50% of miscarriages may be due to the application of the array-based gonome-wide techniques such as single nucleotide polymorphism (SNP) (49). However, the proportion of late miscarriages with chromosomal abnormalities of the fetus is lower than that of early miscarriages, as later pregnancy losses more often occur due to s uterine, cervix uteri insufficiency or immune factors (50, 51). So it is suggesting that the significant higher proportion of early abortion in the inferior-quality group may be due to the high proportion of the euploidy state of the transfer embryos.

There are also some limitations in our study. First, although single good-quality blastocyst-stage embryo transfer can achieve a comparable live birth rate and significantly lower multiple pregnancy rates compared with the double cleavage-stage embryo transfer group, it also increases the transfer cancellation rates because no good-quality blastocyst formation occurs in long-term culture *in vitro*, so the cumulative live birth rates per oocyte pick-up cycle, transfer cancellation rates and duration from oocyte pick-up to clinical pregnancy should be considered, and the maternal pregnancy risk and long-term follow-up studies on infants also need to be considered in the next study. Second, this study is a retrospective analysis, not a randomized controlled study, further prospective studies need to be carried out to verify these findings in multicenter trials with larger numbers of samples. Finally, there are subjective differences in blastocyst morphology scores among different embryologists, which may lead to some bias in the results, a fixed embryologist to evaluate the morphology of the blastocysts in the enrolled couples will eliminate the bias in the results.

In conclusion, based on the current evidence, single good-quality day 6 blastocyst embryo transfer is the optimal strategy for the first warming cycle of the patients who had no day 5 blastocyst cryopreservation, which can achieve a comparable live birth rate and lower the multiple pregnancy risk compared with the double cleavage-stage embryo transfer. our study provided a theoretical foundation for the clinicians and the embryologists to choose the most suitable frozen-thawed transplantation scheme for the patients without day5 blastocyst formation in the fresh cycles.

## Data Availability

The original contributions presented in the study are included in the article/supplementary material. Further inquiries can be directed to the corresponding author.
